# Unlocking access: a comprehensive analysis of medicines accessibility for rare diseases in Thailand

**DOI:** 10.1186/s13023-025-03754-9

**Published:** 2025-05-28

**Authors:** Siriwat Suwattanapreeda, Sanita Hirunrassamee, Chaoncin Sooksriwong, Kusawadee Maluangnon, Thirapich Chuachantra, Krissana Kuchaisit, Niti Osirisakul

**Affiliations:** 1 Somdet Chaopraya Institute of Psychiatry, 112 Somdetchaopraya Street, Klongsarn, Bangkok, 10600 Thailand; 2 Drug Information and Consumer Protection Center, Center of Excellence in Pharmacy Practice and Management Research, Faculty of Pharmacy, Thammasat University, Khlong Luang District, Pathum Thani Thailand; 3 Medicines Regulation Division, Food and Drug Administration, Ministry of Public Health, Nonthaburi, Thailand; 4 Pharmacy Division, Faculty of Medicine Ramathibodi Hospital, Bangkok, Thailand

**Keywords:** Rare diseases, Accessibility, Health insurance coverage, IRDiRC, The national essential medicines list

## Abstract

**Introduction:**

In Thailand, obtaining medicines for rare diseases presents significant challenges, with limited evidence highlighting these issues.

**Objectives:**

To evaluate the accessibility of medicines and the extent of health insurance coverage for treatments of rare diseases in Thailand.

**Method:**

This study utilized a thorough review of current health policies, drug registration database, and insurance coverage conditions. Additionally, procurement data from the Ministry of Finance was analyzed to verify the acquisition of medicines intended for the treatment of rare diseases.

**Results:**

A review of the availability and procurement of medicines for rare diseases in Thailand revealed considerable limitations in both registration and accessibility. According to the International Rare Diseases Research Consortium, only 46.80% of their recommended medicines were registered in Thailand, and of these, just 22.93% were included in the national essential medicines list. Additionally, a review of the state’s pharmaceutical procurement dataset over the past 5 years showed that merely 31.70% of these registered drugs had been purchased from suppliers for use in hospitals.

**Conclusion:**

To address these issues, the study recommended accelerating the approval process for rare disease medicines, expanding health insurance coverage, establishing financial support for patients, and creating a specific pricing policy for orphan drugs. Collaborative efforts among stakeholders were emphasized as crucial for improving access to essential medicines and enhancing treatment outcomes for patients with rare diseases in Thailand.

**Supplementary Information:**

The online version contains supplementary material available at 10.1186/s13023-025-03754-9.

## Introduction

Rare diseases are a category of chronic illnesses that are uncommon and a significant impact on life expectancy or chronic debilitation, with each disease having a prevalence of less than 1 in 2000 individuals in the population. Many rare diseases are known to impact fewer than one person per 100,000 individuals. Around 7000–10,000 rare diseases have been identified, and recent analysis indicates a conservative prevalence rate ranging from 3.5 to 5.9% [1–3].

Rare diseases are conditions that are not common. They make up about 10–25% of chronic diseases in adults. These diseases can be serious and have symptoms that might be life-threatening. Many of them show up in childhood, with about half being noticed shortly after birth [4]. Moreover, 95% of rare diseases currently have no treatment available [5].

In Thailand, there has been no comprehensive survey conducted to determine the prevalence of rare diseases among the population. Furthermore, there is no clear delineation of which diseases are officially categorized as rare diseases in the country. Consequently, it is challenging to estimate the true number of affected individuals [6].

In 2019, the National Health Security Office (NHSO) of Thailand, which administers the largest public health insurance program in the country, identified 24 rare diseases for inclusion in their benefit package. The NHSO adopted a capitation-based payment structure, allocating approximately 212 USD per patient annually (using a 33 THB to 1 USD exchange rate) for diagnostic evaluations. Additionally, the plan provides for capitation payments ranging from about 1515 to 9091 USD per patient each year for confirmatory testing, treatment, and ongoing management [7].

In terms of medical expertise, Thailand had around 20 specialized physicians in 2019 to treat these rare conditions. These specialists were primarily affiliated with seven University hospitals, most of which are situated in Bangkok, the nation’s capital [8].

Currently, Thailand lacks specific policies that address access to medications for treating rare diseases. Additionally, there are no established policies to shield patients with rare diseases from catastrophic financial burdens caused by the high cost of these medications.

According to data from the National Health Security Office (NHSO), patients with rare diseases in Thailand encounter significant challenges in accessing necessary healthcare. These challenges include limited availability of specialized physician, which is primarily provided in university hospitals, and restricted access to their essential medications.

Research on pharmaceutical usage and policy concerning rare diseases in Thailand is scarce. Conducting thorough studies in this area could be highly beneficial, particularly for developing countries that lack adequate pharmaceutical policies for rare diseases. Such research would offer valuable insights and potentially assist these nations in developing strategies to improve access to medical treatments, thereby better supporting their healthcare needs.

This study aimed to evaluate the accessibility of medicines and the extent of health insurance coverage for treatments of rare diseases in Thailand.

## Method

This study employed a cross-sectional design to examine the availability of medicines for the treatment of rare diseases among underserved patient populations. A comprehensive approach was utilized to assess medicine accessibility, focusing on two key dimensions: the presence of medicines within the healthcare system and the ability of patients to obtain them.

To evaluate the existence of medicines, the study conducted a comprehensive document analysis, alongside an examination of procurement data from the Ministry of Finance over the past 5 years.

This process involved reviewing historical medication purchase records to identify procurement patterns and trends, thereby providing insights into the availability of medicines for rare disease treatment.

To assess the ability to obtain medicines, the study explored the benefit packages of public health insurance systems.

*Document analysis*: This component involved reviewing various documents such as governmental publications, legal statutes, and organizational records to understand the broader context of medication access for rare diseases. Relevant documents were collected from reputable sources, including government websites and scholarly databases, based on their potential to provide insights into medication accessibility.

*Procurement data analysis*: Procurement data from the Ministry of Finance was analyzed to validate the purchase of medicines used for treating rare diseases, thereby confirming their availability in government hospitals. This dataset included information such as medication names, quantities purchased, costs, and suppliers. The decision to utilize public sector procurement data was driven by the fact that the diagnosis and treatment of rare diseases and other conditions frequently occur within public healthcare settings.

To assess the existing situation, this study employed a tracer technique as a methodological tool [9], utilizing a list of tracer drug items recommended by the International Rare Diseases Research Consortium (IRDiRC) [10]. These tracers included examining the registration status and labeled indications found in package inserts approved by the Thai Food and Drug Administration (FDA). Furthermore, the study evaluated the inclusion status of these medicines in the National List of Essential Medicines and the benefit packages of health insurance systems to assess both accessibility and coverage provided by health insurance schemes.

All collected data were synthesized to formulate policy recommendations for policymakers.

## Results

Upon reviewing the Thai FDA drug registration list and analyzing the 5 years public hospital procurement data from the Ministry of Finance, the results show in Table 1. Table 1 reveals a comparison information of IRDiRC recommendation on each drug group with the FDA registration data and purchasing data.

**Table 1:** The number of medicines registered and documented for purchase and utilization in hospitals in Thailand

Main disease category	Number of medicines			Medicines registered but not available in public hospitals	
	IRDiRC recommendation	Registration (% of recommendation)	Purchase history (%of registration)	Condition	Drug items
Endocrine	15	8 (53.33%)	7 (87.50%)	Hypoparathyroidism	Parathyroid hormone
Hematologic	42	21 (50.00%)	13 (61.90%)	Hemophilia A	Lonoctocog alfa Emicizumab
				Factor X Deficiency	Human coagulation factor X
				Anemias Sickle Cell Anemia	Hydroxyurea
				Other Hematologic Disorders Congenital And Acquired Methemoglobinemia	Methylene blue injection
				Conditioning For Hematopoietic Stem Cell Transplant	Thiotepa
				Immune (Idiopathic) Thrombocytopenic Purpura	Romiplostim
				Hemophilia (Factor Vii Deficiency)	Recombinant Factor VIIa
Immunologic	3	0 (0.00%)	0 (0.00%)	–	–
Inflammatory	29	17 (58.62%)	12 (70.59%)	Juvenile Rheumatoid Arthritis	Tocilizumab
				Pediatric Ulcerative Colitis	5-aminosalicylic acid
				Juvenile Rheumatoid Arthritis	Golimumab
				Hereditary Angioedema	Lanadelumab
				Hereditary Angioedema	Icatibant acetate
Metabolic	49	20 (40.82%)	11 (55.00%)	N-Acetylglutamate Synthetase Deficiency	Carglumic acid
				Lysosomal Storage Diseases Gaucher Disease	Velaglucerase alfa
				Fabry Disease (Alphagalactosidase A Deficiency)	Agalsidase beta Agalsidase alfa
				Pompe Disease	Alglucosidase alfa
				Mucopolysaccharidosis I (Iduronidase Deficiency)	Laronidase
				Hunter Syndrome (Mucopolysaccharidosis Ii)	Idursulfase
				Metabolic Acidosis	Trisodium citrate
				Wilson Disease	Zinc acetate
Neurologic	34	17 (50.00%)	15 (88.24%)	Transthyretin Amyloidosis	Tafamidis
				Multiple Sclerosis	Siponimod
					Teriflunomide
				Non-Dystrophic Myotonic Disorders	Mexiletine HCl
				Parkinson Disease (Young And Early-Onset)	Selegiline
				Amyotrophic Lateral Sclerosis	Riluzole
Pulmonary	24	10 (41.67%)	6 (60.00%)	Pulmonary Arterial Hypertension	Selexipag Iloprost
				Idiopathic Pulmonary Fibrosis	Pirfenidone
Miscellaneous	9	2 (22.22%)	1 (50.00%)	Autosomal Dominant Polycystic Kidney Disease	Tolvaptan

The number of drugs registration should reflex the availability or existence and information of purchasing history should reflex their accessibility. The number of registered drugs in each group are higher than purchasing. Somehow, it means that there are many drugs ready to be purchased but were not in need.

A. Availability of medicines for the treatment of rare diseases

It was found that in almost drug group, there were drugs registered, or available. But not all the drugs items listed as IRDiRC recommendation are registered. The top 3 highest available percentage were drugs for Inflammatory (58.62%), Endocrine (53.33%), Hematologic (50.00%), Neurologic (50.00%) and Pulmonary (41.67%) while the drug for immunologic was not registered and not available.

B. Accessibility of medicines for the treatment of rare diseases

Only the drugs that were purchased would be accessed. The number of drugs purchased were less than the number registered. The top most purchased were drugs for Neurologic, 15 items purchased from 17 registered or 88.24%; Endocrine, 7 items purchased from 8 registered or 87.5%; and Inflammatory, 12 items purchased from 17 registered or 70.59%. It should be noted that the drugs for Inflammatory group which was the top registered was not the top purchased. There were 11 items purchased from 17 registered or only 64.71%. The rest were purchased at the rate of 61.90%, 60.00%, 55.00%, 50.00%, and 0% of availability. So, it can be concluded that there were drugs available but the purchasing would depend on the need of treatment. There were drugs available for conditions listed in the Table 1, but never be purchased.

C. Analysis of the barriers

Upon reviewing policies and measures originated from the Ministration of Public Health, it was found that there were issues related to the national drug policies, the National List of Essential Medicines, the benefit package of health insurance, and drug price policies that might hinder registration of drugs for treatment of rare diseases. (See Supplement 1).

1. National drug policy

In 1981 (B.E. 2524), Thailand initiated the first National Drug Policy overseen by the Minister of Public Health. Currently, the country is implementing its forth version of the National Drug Policy, which spans from B.E. 2563 to B.E. 2565. All editions of the National Drug Policy did not explicitly address the challenge of medication accessibility for individuals with rare diseases, as they were formulated with a broader scope. Nonetheless, they included certain indicators or strategy that indirectly impacted medication access for these patients.

1. From 1993 to 2011 (B.E. 2536–2554), there was a pattern of inconsistent policy due to the frequent turnover of the National Drug Committee caused by parliamentary dissolution. To address this issue, the Regulations of the Office of the Prime Minister on National Drug System Development Committee B.E. 2551 were established in 2008. During that same year, the committee introduced the NED (E 2) policy to address the challenge of accessing high-priced medicines. This policy facilitated public access to essential medicines, including those for certain rare diseases [11].
2. The 4 th National Drug Policy, spanning from B.E. 2563 to B.E. 2565, includes indicators aimed at ensuring continuous access to essential medicines for Thai population. The specified medication list encompasses essential national drug lists [as defined in Sub-list E (2)] and orphan drugs, some of which cater to patients with rare diseases [12].
3. Essential drug list

The National List of Essential Medicines in Thailand was first established in 1996 with the objective of encompassing a list of necessary medicines to address the country’s public health issues [13]. Medicines included in the National List of Essential Medicines must be registered with the Food and Drug Administration (FDA), and the National List of Essential Medicines does not cover off-label use.

Regarding drugs listed in the National Essential Drug List: In cases where off-label uses are supported by clear evidence and are internationally recognized, even if not registered in Thailand, they may still be considered. Approval from the National Drug List Development Committee is required [14].

Presently, the National List of Essential Medicines serves as a tool within the public health insurance system, functioning as a referenced pharmaceutical benefit package for accessing essential medicines [15].

Medicines listed in the National List of Essential Medicines are accessible to individuals who are entitled to public health insurance provided by the government. These individuals receive supported medication expenses from the government, thus eliminating the need for out-of-pocket payments. However, it is a requirement that the medication is used for the treatment of conditions supported by the National List of Essential Medicines, and that it is obtained from healthcare facilities participating in the government’s health insurance program.

(3) The extent of health insurance coverage for treatments of rare diseases

In Thailand, the government has established three key health insurance systems aimed at providing coverage to the entire Thai population. These systems include the Civil Servant Medical Benefit Scheme, the Social Security Scheme, and the Universal Health Coverage Scheme. (See Supplement 1).

### Civil servant medical benefit scheme (CSMBS)

The CSMBS is designed to cater to government employees and their dependents (6% of the total population) are covered by a tax-financed non-contributory Civil Servant Medical Benefit Scheme (CSMBS) as a fringe benefit managed by the Comptroller General, Department of the Ministry of Finance. It offers comprehensive healthcare coverage, encompassing outpatient, inpatient, and specialized services [16].

### Social security scheme (SSS)

Targeting private sector employees (excluding dependents) (19% of the total population), the SSS provides healthcare coverage funded by contributions from three parties: employers, employees, and the government.

### Universal health coverage scheme (UHC)

The UHC is the cornerstone of Thailand’s healthcare welfare system, aiming to provide coverage to uninsured and vulnerable populations (75% of the population). It is financed through general taxation and managed by the National Health Security Office [17].

For the basic drug benefit packages of all three insurance schemes, they are similar in that they cover medicines listed in the National List of Essential Medicines.

Notwithstanding, there exists a distinction in the Civil Servant Medical Benefit Scheme (CSMBS) regarding its coverage, which includes medicines not listed on the National List of Essential Medicines, contingent upon their prescription according to the indications approved by the Food and Drug Administration (FDA). An exception is made for medicines classified under the categories of high-cost drugs, and/or biological products, and/or targeted therapies that were registered after 1 January 2018. Such medicines are subject to evaluation by the Comptroller General’s Department of the Ministry of Finance to determine eligibility for reimbursement. For instance, Lanadelumab, used in the treatment of Hereditary Angioedema, is not included in the CSMBS benefit package [18].

Due to the exclusion from the national list of essential medicines and classification as high-cost and/or biological products and/or targeted therapies, many indispensable drugs for the treatment of rare diseases face significant barriers as they do not meet the criteria for inclusion in the benefits package. Additionally, even when a medication is approved by the Thai Food and Drug Administration (FDA), its use in an off-label manner further excludes it from the benefits package criteria. This is particularly relevant for rare diseases, where off-label prescription is common due to limited data on drug usage. The scarcity of patient populations may preclude the registration or specific indication labeling of such drugs [19]. For instance, pasireotide, despite being an unlisted essential medicine with FDA-approved indications for the treatment of Cushing’s disease [20], is not included in the Civil Servant Medical Benefit Scheme (CSMBS) benefits package for the treatment of Acromegaly, showcasing the challenges in accessing treatments for rare conditions within the existing health benefits framework [21].

For the Universal Health Coverage Scheme, if medicines to treat rare diseases aren’t included in the National List of Essential Medicines, they aren’t covered. In Thailand, there isn’t a specific policy for rare diseases. This creates difficulties for patients in accessing the healthcare services and medicines they require [22].

In 2019, the National Health Security Office (NHSO) started managing system for rare diseases and identified 24 of them. The NHSO and Government Pharmaceutical Organization (GPO) also made sure medicines for these diseases are available. The Food and Drug Administration included these medicines in a special list in the National List of Essential Medicines (the drug category E2) and/or Orphan Drugs lists to help patient with rare disease access them easily [22].

For the Social Security Scheme, there has been no additional policy implementation to facilitate access to medicines for rare diseases.

(4) Drug price policy

In the domain of rare diseases, Thailand presently lacks a designated pricing policy. However, within the country, there exists a system of price control governing medication procurement by government-owned hospitals. This system imposes ceiling prices to ensure hospitals do not procure medicines at rates surpassing those officially declared by the government. These officially declared prices, known as Reference Prices for Public Procurement, encompass nearly all drugs deemed essential and listed in the National List of Essential Medicines.

(5) Market availability of tracer drug for rare disease in Thailand

Based on the survey conducted on the drug registry for rare diseases, it has been determined that Thailand maintains a list of medicines for rare diseases, comprising 95 items, which represents 46.80 percent of those recommended by the International Rare Diseases Research Consortium (IRDiRC), as depicted in supplementary. Among these medicines, only 47 items (22.93%) are included in the National List of Essential Medicines.

An analysis of the prescribing guidelines from the national essential medicines list shows that the treatment indications for many drugs intended for rare diseases are not adequately covered. This lack of comprehensive coverage prevents patients insured through the NHSO and SSS from accessing essential treatments under the assured financial protection of these programs. Consequently, these patients are unable to access these crucial medicines with guaranteed cost coverage, highlighting a critical gap in the provision of healthcare services and the need for policy enhancement to better accommodate the needs of those with rare diseases. For instance, Octreotide acetate injection is listed for specific conditions such as (1) treatment for high output pancreatic fistula, (2) adjunct treatment for variceal bleeding alongside therapeutic endoscopic intervention, (3) treatment for bleeding associated with portal hypertensive gastropathy, and (4) treatment for neuroendocrine tumors. However, for treating Acromegaly—a rare disease—Octreotide acetate does not meet the list’s indications, which restricts patient access to this important drug under the specified insurance conditions.

Furthermore, only 31.70% of the recommended medicines have a documented purchase history. The list of registered medicines in Thailand is available in the supplementary materials.

Although medicines for the treatment of rare diseases have been officially registered, there is no evidence from government procurement data over the past 5 years that these medicines have been acquired for hospital use. This lack of procurement may be due to the absence of diagnosed patients with these rare diseases or because the costs of treatment are not covered by the health insurance system, as shown in Table 1. However, the registration of these drugs may indicate the existence of patients with these rare diseases in the country.

#### Synthesis the unlocking to access

Empirical findings derived from tracer medicine lists indicate that the management system for rare disease medicines requires significant enhancement to improve accessibility. Policymakers can achieve this through the implementation of five strategic pillars aimed at facilitating access to these crucial medicines.

In countries lacking dedicated healthcare systems for rare diseases, such as Thailand, it is essential that health insurance protection measures do not obstruct access and affordability, ensuring that individuals receive the necessary care. To increase access to medicines, a comprehensive approach employing various strategies is imperative. These strategies may include.

1. Regulatory Pathway:

Although the national drug policy promotes access to essential medicines, in practice, the registration process for drugs treating rare diseases closely resembles that of conventional medicines. Furthermore, when examining the number of drug listings for rare diseases, only 49.75% of the recommended medicines (according to IRDiRC) are actually available. Moreover, there is a rigidity in adhering to the criteria for approving the label use of drugs for rare diseases within the regulatory framework. Therefore, it is imperative to establish a fast-track pathway for registering new medicines intended for rare diseases. Additionally, a distinct fast-track process with specific criteria for evaluating approvals should be implemented for label use in rare diseases. This approach should diverge from the standard process for drugs treating common diseases, thereby facilitating pharmaceutical companies in expediting the introduction of these medicines to the market.

2. Coverage with Specific Conditions:

Under the benefit packages of health insurance systems like NHSO and SSS, coverage is only provided for drugs included in the national essential medicines list. This limitation means that only a few treatments for rare diseases are covered, leaving insured patients unable to access other necessary medicines without having to bear the costs themselves. Moreover, if the national essential medicines list does not specifically mention the indications for a rare disease, patients are required to pay out of pocket.

Therefore, expanding health benefit coverage under public health insurance schemes for the treatment of rare diseases, allowing the utilization of non-national essential medicine lists, and/or essential medicine including off-label medicines and special indication, is crucial.

3. Safety Net for Catastrophic Protection:

Due to budget constraints, the healthcare system, including both the Universal Health Coverage Scheme (UHC) and Social Security Scheme (SSS), is unable to cover all medical expenses in treatment of rare disease. These schemes operate on a capitation payment method for hospitals, limiting access to medication to those listed in the benefit package, primarily essential national drug list items. However, the lack of coverage for non-essential drugs poses a significant limitation. To address this issue and alleviate financial burdens for patients with rare diseases, implementing financial assistance measures in the form of a Safety Net is crucial. This safety net would offer full financial coverage for healthcare expenses to patients with rare diseases who surpass a certain out-of-pocket threshold.

4. Funding Support:

The healthcare system should not leave anyone behind, especially those with rare diseases. Health insurance schemes should not prioritize treating only common illnesses that affect the majority of the population. Everyone should have the right to access healthcare services, even if they are expensive. To achieve the objective, budgets from the three public health insurance schemes should be allocated to create a dedicated fund for the care and treatment of rare diseases.

5. Financial Incentives or Special Conditions for Pharmaceutical Companies:

In Thailand, there is no pricing policy in place for medicines. Instead, the only pricing mechanism enforced is the “reference pricing” policy implemented during drug procurement. The absence of price control measures can impact the affordability of medicines for patients, especially when they have to bear the treatment costs themselves. Moreover, it affects the budget constraints of the healthcare insurance system if it is responsible for covering expenses.

To address this issue, implementing mechanisms to negotiate or regulate drug prices for rare diseases during the drug registration process could be beneficial. This might entail providing tax incentives or benefits to pharmaceutical companies that produce medicines for rare diseases. By doing so, it could help mitigate the financial burden on patients and ensure better access to essential medicines without straining the healthcare system’s budget.

## Discussion

From the list of medicines recommended by the IRDiRC, overall about 50% of the medicines listed by the IRDiRC are registered in Thailand. Therefore, to increase the accessibility of medications for patients, Thailand should allow these medicines to be registered beforehand when it is found that there are patients who need such drugs. They can be imported to meet treatment needs urgently without having to wait for registration process.

Insights from successful international practices demonstrate that many countries have adopted Accelerated Development and Market Access Measures to improve drug accessibility. Notable examples include:

**The United States**

The United States has established several programs designed to expedite the time required for new drugs to reach the market. Key initiatives include Priority Review and Accelerated Approval, both introduced in 1992, as well as the Breakthrough Therapy Designation, launched in 2012. These programs aim to streamline the drug development and approval processes, thereby facilitating faster access to innovative treatments [23].

**The European Union (EU)**

The European Union has implemented similar measures to expedite drug approval and availability. Significant programs include Accelerated Assessment and Conditional Marketing Authorization, both initiated in 2004, and PRIME (Priority Medicines), launched in 2018. These initiatives are designed to reduce regulatory review timelines and ensure more rapid access to essential medicines for patients [23].

**Japan**

In Japan, regulatory initiatives such as the Sakigake (Forerunner) Review, introduced in 2014, and the Orphan Drug Designation have been developed to provide special privileges. These programs shorten review and approval timelines, enabling faster market access for drugs intended to treat critical and rare medical conditions [24].

By adopting similar measures, Thailand could significantly improve access to essential medicines for patients with urgent and unmet needs. Such strategies would align with international best practices and contribute to addressing the challenges of rare disease treatment in the country.

In cases where a pharmaceutical company imports these medicines in advance for stockpiling for the treatment of patients with rare diseases, when there is a need, the government should have measures to compensate for the loss due to medicines expiration to ensure fairness and to motivate companies to stockpile the medicines. This will increase the accessibility of medicines for patients. Compensation measures may be implemented through tax measures or others, not necessarily in the form of monetary compensation.

In general, many countries employ two primary strategies to enhance the accessibility of medicines: first, empowering patients and their communities [25], and second, boosting research and development efforts [25, 26] Thailand has also adopted these strategies. Empowering patients involves giving them and their communities more control over health decisions, which is crucial for better disease management. Although Thailand has attempted to implement this strategy, the results remain inconclusive.

The second strategy, promoting ongoing research and development, aims to garner more governmental support for rare disease research and foster collaborative scientific studies to enhance our understanding of these diseases. However, Thailand faces significant challenges in fully implementing these strategies due to developmental hurdles.

Nevertheless, insights from countries with well-established frameworks for fostering pharmaceutical innovation—particularly in advanced areas such as gene therapy, which Thailand has the potential to develop—underscore the importance of targeted financial, regulatory, and marketing policies.

### Financial incentives for orphan drug development

Financial incentives are vital for encouraging pharmaceutical innovation, particularly in the development of orphan drugs. Various countries have implemented measures such as tax credits, research and development grants, and waivers for regulatory fees to reduce the financial burden on developers, thereby fostering greater investment in this critical area.

**The United States**

The Orphan Drug Act (ODA), enacted in 1983, provides robust financial incentives to pharmaceutical companies. Under this legislation, the Food and Drug Administration (FDA) offers the Orphan Drug Designation, which includes benefits such as tax credits for clinical trials, waivers of regulatory fees, and an extended 7-year market exclusivity for approved orphan drugs. These measures have significantly increased the availability of treatments for rare diseases, effectively encouraging investment and innovation in this essential healthcare domain [27].

**The European Union (EU)**

The European Union has implemented comprehensive measures to support R&D for orphan drugs, including initiatives such as Horizon Europe (2021–2027). Moreover, the Council of the European Union adopted Regulation (EC) No. 141/2000, which incentivizes pharmaceutical companies to develop treatments for rare diseases. Since the adoption of this regulation, the European Medicines Agency (EMA) has approved over 150 orphan drugs, compared to only eight prior to the regulation. This progress underscores the critical role of financial and regulatory support in advancing drug development for rare diseases [28, 29].

**Japan**

Japan has developed a comprehensive framework of incentives to promote orphan drug development. Financially, the National Institute of Biomedical Innovation (NIBIO) offers subsidies to offset R&D costs, and developers can claim a 12% tax credit on eligible expenditures. Regulatory incentives include priority consultations and expedited reviews by the Pharmaceuticals and Medical Devices Agency (PMDA). Additionally, orphan drugs benefit from reduced clinical data requirements and are eligible for inclusion in the National Health Insurance (NHI) system, ensuring financial accessibility for patients [30].

**Australia**

Australia employs similar strategies to support orphan drug development. The Therapeutic Goods Administration (TGA) waives or reduces fees for orphan drug applications, including application and evaluation fees. Regulatory support includes expedited assessments and enhanced guidance for developers. Marketing incentives provide 5 years of market exclusivity, ensuring a protected market for developers. Furthermore, orphan drugs may be listed on the Pharmaceutical Benefits Scheme (PBS), which subsidizes costs, improving affordability for patients [30].

**Policy Implications for Thailand**

The strategies implemented in these countries emphasize the importance of integrating comprehensive financial, regulatory, and marketing support to promote orphan drug development. By adopting similar measures, Thailand could create a more supportive environment for rare disease treatment development. Such incentives would not only foster innovation but also ensure timely and equitable access to essential medications. This, in turn, would address unmet medical needs and improve the quality of life for patients with rare diseases. These international models provide a roadmap for Thailand to overcome existing barriers and establish a robust framework for advancing pharmaceutical innovation.

Additionally, strategies such as negotiating lower drug prices for treating rare diseases are logical. However, in countries like Thailand that lack a drug pricing policy, there could be challenges in effectively implementing this strategy [22, 23].

Despite the finding that approximately 50% of the IRDiRC-recommended medicines are registered in Thailand, further efforts are needed to strengthen the country’s policies. By adopting financial incentives and accelerated approval measures, Thailand could significantly enhance drug accessibility and better address the unmet medical needs of patients with rare diseases. These international models provide a roadmap for Thailand to overcome existing challenges and advance pharmaceutical innovation.

While this study may initially seem to focus solely on describing the availability of medicines for rare diseases in Thailand, it offers a substantial methodological contribution, particularly relevant for developing countries with limited capacity to monitor and report indicators related to the availability of rare disease treatments. The study introduces a practical and straightforward approach for assessing and reporting the national-level availability of these medicines. Moreover, this method holds potential for development into a standardized indicator for international benchmarking. Given the absence of a universally recognized metric to measure the availability of orphan drugs, this study addresses a significant gap in global health assessment and provides a foundation for more comprehensive international comparisons.

Nevertheless, using tracer medicines to evaluate accessibility, as demonstrated in this study, provides a clear reflection of existing problems. This approach can facilitate policymakers in designing effective policies.

## Conclusion

This study highlights critical challenges in the accessibility of medicines for rare diseases in Thailand, particularly among underserved patient populations. The findings reveal gaps in medicine availability within the healthcare system and barriers that limit patients’ ability to obtain essential treatments.

To address these issues, the study recommends accelerating the approval process for rare disease medicines, expanding health insurance coverage, establishing financial support mechanisms for patients, and developing a specific pricing policy for orphan drugs to enhance affordability and accessibility. Furthermore, strengthening collaborative efforts among policymakers, healthcare providers, pharmaceutical companies, and patient advocacy groups is essential to creating a more sustainable and equitable rare disease healthcare framework.

By implementing these strategies, Thailand can improve access to life-saving treatments, reduce the economic burden on affected patients and families, and ultimately enhance health outcomes for individuals living with rare diseases. Future research should explore longitudinal assessments of policy interventions and cross-country comparisons to further refine strategies for ensuring equitable access to orphan drugs on a broader scale.

## Supplementary Information

Supplementary material 1

## Data Availability

Data sets and materials for information in this manuscript can be provided by the first author upon reasonable request.

## Abbreviations

IRDiRC: The International Rare Diseases Research Consortium
NHSO: The National Health Security Office
UHC: Universal Health Coverage Scheme
SSS: Social Security Scheme
CSMBS: The Civil Servant Medical Benefit Scheme
