# Supplementary material for: Unlocking access: a comprehensive analysis of medicines accessibility for rare diseases in Thailand
Source: Orphanet J Rare Dis. 2025 May 28;20:258. doi: 10.1186/s13023-025-03754-9 (PMC12121248; doi:10.1186/s13023-025-03754-9)
Supplement: Supplementary file 1 — Supplementary material 1 [file 13023_2025_3754_MOESM1_ESM.docx]

**Supplement S1:**

|  | Condition | Drug | ED status | Health insurance Coverage | | | Purchasing price control |
| --- | --- | --- | --- | --- | --- | --- | --- |
|  |  |  |  | CSMBS | SSS | UC |  |
| Endocrine | | | | | | | |
|  | Growth hormone deficiency in children | Somatropin for injection | NED | 🗸 | X | X | **🗸** |
|  | Acromegaly | Octreotide | E2 | 🗸 | 🗸 | 🗸 | **🗸** |
|  | Acromegaly | Lanreotide | NED | 🗸 | X | X | **🗸** |
|  | Endogenous Cushing’s syndrome | Ketoconazole | D | 🗸 | **🗸** | 🗸 | **🗸** |
|  | Adrenal insufficiency | Hydrocortisone | A | 🗸 | **🗸** | 🗸 | **🗸** |
|  | Paget’s disease (osteitis deformans) | Calcitonin-human for injection (salmon synthetic) | D | 🗸 | **🗸** | 🗸 | X |
|  | Hypoparathyroidism | Parathyroid hormone | NED | 🗸 | X | X | X |
|  | Idiopathic Hypogonadotropic Hypogonadism | Gonadotropin-releasing hormone | NED | 🗸 | X | X | **🗸** |
| Hematologic | | | | | | | |
|  | Hemophilia A (Factor VIII deficiency) | Lonoctocog alfa | NED | 🗸 | X | X | 🗸 |
|  | Hemophilia A (Factor VIII deficiency) | Emicizumab | NED | 🗸 | X | X | X |
|  | Hemophilia A (Factor VIII deficiency) | Desmopressin acetate | D | 🗸 | X | X | 🗸 |
|  | von Willebrand disease | Factor VIII/ von Willebrand factor | E2 | 🗸 | 🗸 | 🗸 | 🗸 |
|  | Hemophilia B (Factor IX deficiency | Human coagulation factor IX | E2 | 🗸 | X | X | 🗸 |
|  | Factor X deficiency | Human coagulation factor X | NED | 🗸 | X | X | 🗸 |
|  | Anemias Sickle cell anemia | Hydroxyurea | C | 🗸 | 🗸 | 🗸 | 🗸 |
|  | Anemia of end-stage renal disease | Epoetin alfa | E2 | 🗸 | 🗸 | 🗸 | 🗸 |
|  | Idiopathic thrombocytopenic purpura, Aplastic anemia | Eltrombopag | NED | 🗸 | X | X | 🗸 |
|  | Beta thalassemia major *(transfusion dependence)* | Deferasirox | E2 | 🗸 | 🗸 | 🗸 |  |
|  | Other Hematologic  Disorders Congenital and acquired methemoglobinemia | Methylene blue injection | D | 🗸 | 🗸 | 🗸 | X |
|  | Essential thrombocythemia | Anagrelide hydrochloride | NED | 🗸 | X | X | 🗸 |
|  | Conditioning for hematopoietic stem cell transplantation | Busulfan | C | 🗸 | 🗸 | 🗸 | 🗸 |
|  | Conditioning for hematopoietic stem cell transplantation | Thiotepa | NED | X | X | X | X |
|  | Iron overload | Deferiprone | C | 🗸 | 🗸 | 🗸 | 🗸 |
|  | Immune (idiopathic) thrombocytopenic purpura | Romiplostim | NED | 🗸 | X | X | X |
|  | Polycythemia vera | Ruxolitinib | NED | 🗸 | X | X | 🗸 |
|  | Hemophilia B (Factor IX deficiency | Nonacog alpha | E2 | 🗸 | 🗸 | 🗸 | X |
|  | Hemophilia (Factor VII deficiency) | Eptacog alpha (activated) | NED | 🗸 | X | X | 🗸 |
|  | Hemophilia (Factor VII deficiency) | Recombinant Factor VIIa | NED | 🗸 | X | X | 🗸 |
|  | Agammaglobulinemia | Immunoglobulin infusion | E2 |  |  |  |  |
| immunologic | | | | | | | |
| Inflammatory | | | | | | | |
|  | Rheumatoid Arthritis  Juvenile rheumatoid arthritis | Methotrexate | C | 🗸 | 🗸 | 🗸 | 🗸 |
|  | Juvenile rheumatoid arthritis | Etanercept | NED | 🗸 | X | X | 🗸 |
|  | Juvenile rheumatoid arthritis | Methylprednisolone | C | 🗸 | 🗸 | 🗸 | X |
|  | Juvenile rheumatoid arthritis | Adalimumab | E2 | 🗸 | 🗸 | 🗸 | 🗸 |
|  | Juvenile rheumatoid arthritis | Tocilizumab | E2 | 🗸 | 🗸 | 🗸 | 🗸 |
|  | Juvenile rheumatoid arthritis | Abatacept | NED | 🗸 | X | X | 🗸 |
|  | Juvenile rheumatoid arthritis | Golimumab | NED | X | X | X | 🗸 |
|  | Gastrointestinal Inflammation Pediatric Crohn’s disease | Adalimumab | E2 | 🗸 | 🗸 | 🗸 | 🗸 |
|  | Pediatric ulcerative colitis | 5-aminosalicylic acid (Mesalazine) | C | 🗸 | 🗸 | 🗸 | X |
|  | Pediatric ulcerative colitis | Adalimumab | E2 | 🗸 | 🗸 | 🗸 | 🗸 |
|  | Hereditary angioedema | Lanadelumab | NED | X | X | X | X |
|  | Hereditary angioedema | Danazol | NED | 🗸 | X | X | 🗸 |
|  | Hereditary angioedema | Tranexamic acid | A,C | 🗸 | 🗸 | 🗸 | X |
|  | Other Inflammatory Disorders  Multiple sclerosis, Behcet’s disease, Familial Mediterranean fever | Colchicine | A | 🗸 | 🗸 | 🗸 | 🗸 |
|  | Anti-neutrophil vasculitis, Wegener’s granulomatosis, ChurgStrauss Syndrome | Rituximab | E2 | 🗸 | 🗸 | 🗸 | 🗸 |
|  | Non-infectious uveitis | Dexamethasone | A | 🗸 | 🗸 | 🗸 | 🗸 |
|  | Juvenile rheumatoid arthritis | Infliximab | E2 | 🗸 | 🗸 | 🗸 | 🗸 |
|  | Pediatric Crohn’s disease | Infliximab | E2 | 🗸 | 🗸 | 🗸 | 🗸 |
|  | Pediatric ulcerative colitis | Infliximab | E2 | 🗸 | 🗸 | 🗸 | 🗸 |
|  | Hereditary angioedema | Icatibant acetate | NED | 🗸 | X | X | X |
|  | Vernal keratoconjunctivitis | Ciclosporin | C | 🗸 | 🗸 | 🗸 | 🗸 |
| Metabolic | | | | | | | |
|  | N-acetylglutamate synthetase deficiency | Carglumic acid | E2d | 🗸 | X | 🗸 | X |
|  | Lysosomal Storage Diseases Gaucher disease | Velaglucerase alfa | NED | X | X | X | X |
|  | Lysosomal Storage Diseases Gaucher disease | Imiglucerase | E2 | 🗸 | X | X | X |
|  | Fabry disease (alphagalactosidase A deficiency) | Agalsidase beta | NED | 🗸 | X | X | X |
|  | Fabry disease (alphagalactosidase A deficiency) | Agalsidase alfa | NED | 🗸 | X | X | X |
|  | Pompe disease | Alglucosidase alfa | NED | 🗸 | X | X | X |
|  | Mucopolysaccharidosis I (Iduronidase deficiency) | Laronidase | NED | 🗸 | X | X | X |
|  | Hunter syndrome (Mucopolysaccharidosis II) | Idursulfase | NED | 🗸 | X | X | X |
|  | Cholesterol, Lipid, Fatty Acid Disorders  Homozygous familial hypercholesterolemia | Evolocumab | NED | 🗸 | X | X | 🗸 |
|  | Cholesterol, Lipid, Fatty Acid Disorders  Homozygous familial hypercholesterolemia | Rosuvastatin calcium | NED | 🗸 | X | X | 🗸 |
|  | Cholesterol and bile acid synthesis defects | Cholic acid | NED | 🗸 | X | X | X |
|  | Hypophosphatemic rickets (x-Linked) | Burosumab-twza | NED | X | X | X | X |
|  | Hyperphosphatemia in renal failure | Calcium acetate | NED | X | X | X | X |
|  | Osteogenesis imperfecta | Alendronate | D | 🗸 | 🗸 | 🗸 | 🗸 |
|  | Scurvy | Ascorbic acid | A | 🗸 | 🗸 | 🗸 | X |
|  | Metabolic acidosis | Thiamine | A | 🗸 | 🗸 | 🗸 | 🗸 |
|  | Metabolic acidosis | Trisodium citrate | NED | 🗸 | X | X | X |
|  | Prevention of uric acid nephrolithiasis | Potassium citrate | A | 🗸 | 🗸 | 🗸 | X |
|  | Wilson disease | Penicillamine | C | 🗸 | 🗸 | 🗸 | X |
|  | Wilson disease | Zinc acetate | NED | 🗸 | X | X | X |
| Neurologic | | | | | | | |
|  | Transthyretin amyloidosis | Tafamidis | NED | X | X | X | X |
|  | Parkinson Disease (Young and Early-onset) | Rasagiline | NED | 🗸 | X | X | 🗸 |
|  | Parkinson Disease (Young and Early-onset) | Selegiline | NED | 🗸 | X | X | 🗸 |
|  | Parkinson Disease (Young and Early-onset) | Pramipexole | NED | 🗸 | X | X | 🗸 |
|  | Dystonia, Spasticity | Baclofen | B | 🗸 | 🗸 | 🗸 | 🗸 |
|  | Tuberous Sclerosis complex | Everolimus | D | 🗸 | X | X | 🗸 |
|  | Spina bifida (prevention) | Folic acid | A | 🗸 | 🗸 | 🗸 | 🗸 |
|  | Biotinidase deficiency | Biotin | NED | 🗸 | X | X | X |
|  | Epilepsy Infantile spasms | Vigabatrin | D | 🗸 | 🗸 | 🗸 | 🗸 |
|  | Status epilepticus | Midazolam | D | 🗸 | 🗸 | 🗸 | 🗸 |
|  | Juvenile myoclonic epilepsy, Generalized epilepsy | Levetiracetam | C,D | 🗸 | 🗸 | 🗸 | 🗸 |
|  | Complex and rare disease epilepsy | Clobazam | NED | 🗸 | X | X | X |
|  | Complex and rare disease epilepsy | Lamotrigine | C | 🗸 | 🗸 | 🗸 | 🗸 |
|  | Complex and rare disease epilepsy | Topiramate | D | 🗸 | 🗸 | 🗸 | 🗸 |
|  | Neuromuscular Diseases  Amyotrophic lateral sclerosis | Gabapentin | D | 🗸 | 🗸 | 🗸 | 🗸 |
|  | Amyotrophic lateral sclerosis | Riluzole | NED | 🗸 | X | X | X |
|  | Myasthenia gravis | Pyridostigmine Bromide | A | 🗸 | 🗸 | 🗸 | 🗸 |
|  | Multiple Sclerosis | teriflunomide | NED | X | X | X | 🗸 |
|  | Multiple Sclerosis | Fingolimod HCl | NED | 🗸 | X | X | 🗸 |
|  | Multiple Sclerosis | Siponimod | NED | 🗸 | X | X | X |
|  | Parkinson Disease (Young and Early-onset) | Carbidopa/Levodopa | A | 🗸 | 🗸 | 🗸 | 🗸 |
| Pulmonary | | | | | | | |
|  | Pulmonary arterial hypertension | Macitentan | NED | 🗸 | X | X | 🗸 |
|  | Pulmonary arterial hypertension | Sildenafil | D | 🗸 | 🗸 | 🗸 | 🗸 |
|  | Pulmonary arterial hypertension | Bosentan monohydrate | NED | 🗸 | X | X | 🗸 |
|  | Pulmonary arterial hypertension | Selexipag | NED | X | X | X | 🗸 |
|  | Pulmonary arterial hypertension | Iloprost | NED | 🗸 | X | X | 🗸 |
|  | Idiopathic Pulmonary Fibrosis | Pirfenidone | NED | 🗸 | X | X | X |
|  | Idiopathic Pulmonary Fibrosis | Nintedanib | NED | 🗸 | X | X | 🗸 |
|  | Primary apnea of premature newborns | Caffeine citrate | NED | 🗸 | X | X | X |
|  | Lymphangioleiomyomatosis, Tuberous sclerosis | Sirolimus | D | 🗸 | X | X | 🗸 |
|  | Pulmonary arterial hypertension | Tadalafil | NED | X | X | X | 🗸 |
| Miscellaneous | | | | | | | |
|  | Ventricular tachycardia | Amiodarone | C | 🗸 | 🗸 | 🗸 | 🗸 |
|  | Autosomal dominant polycystic kidney disease | Tolvaptan | NED | 🗸 | X | X | 🗸 |

Note: Accessed all related data on July 21, 2024
